# High gravity material extrusion system and extruded polylactic acid performance enhancement

**DOI:** 10.1038/s41598-023-40018-7

**Published:** 2023-08-30

**Authors:** Xin Jiang, Ryo Koike

**Affiliations:** 1 Research and Development Department, Kanagawa Institute of Industrial Science and Technology, 705-1 Shimoimaizumi, Ebina, Kanagawa 243-0435 Japan; 2 Department of System Design Engineering, Keio University, 3-14-1, Hiyoshi, Kohoku-ku, Yokohama, Kanagawa 223-8522 Japan

**Keywords:** Mechanical engineering, Design, synthesis and processing, Polymers

## Abstract

Additive manufacturing (AM) has gained significant attention in recent years owing to its ability to quickly and easily fabricate complex shapes and geometries that are difficult or impossible to achieve with traditional manufacturing methods. This study presents the development of a high-gravity material extrusion (HG-MEX) system, which generates a high-gravity field through centrifugal acceleration. In this process, the material is dissolved by heating the nozzle and subsequently deposited on the construction platform. The primary objective of this research is to evaluate the positive effects of gravity on material extrusion (MEX), which is a key aspect of AM. To accomplish this, a combined machine comprising a MEX unit and centrifuge is constructed. This HG-MEX system is used to analyze and reflect the influence of gravity on the material extrusion. The experimental evaluations demonstrate that the application of high gravity is a promising approach to improve the shape accuracy and performance of the parts fabricated through MEX. Notably, our results confirm the feasibility of utilizing MEX under high gravity to enhance performance in AM processes.

## Introduction

Additive manufacturing (AM) is typically employed for creating three-dimensional objects by sequentially adding layers of material^1^. Unlike traditional manufacturing methods, AM can easily fabricate complex shapes and geometries that are otherwise challenging or unfeasible^2–5^. Furthermore, the range of materials used in AM is diverse, encompassing plastics, metals, ceramics, and even biological materials^6–10^. Consequently, AM has a wide range of applications in industries such as aerospace, automotive and healthcare^11^. Thus, AM holds the potential to revolutionize manufacturing by enabling faster, more efficient, and more customized production.

A notable characteristic of AM is its ability to facilitate the flexible fabrication of complex shapes and individual design changes^12^. Sehhat et al.^13,14^ studied the effect of temperature and material variation on mechanical properties of parts fabricated via fused deposition modeling (FDM). Additionally, they validated stress transformation in anisotropic material additively manufactured via FDM. Moreover, Mohamed et al.^15^ studied the optimization of FDM process parameters. Furthermore, Charalampous et al.^16^ investigated machine learning-based mechanical behavior optimization of 3D-printed constructs manufactured via the FFF process. In addition, Li et al.^17^ studied the effect of process parameters in fused deposition modelling on bonding degree and mechanical properties. AM is expected to become the core technology of the next-generation manufacturing system^18–20^. In the 2010s, NASA initiated 3D printing tests in the International Space Station, aiming to ensure sustainability in various space activities^21^. From 2020 onward, the long-term projects involving lunar and Martian missions necessitate maintenance and repair capabilities in regulated space environments. In this regard, AM has garnered significant attention because of its excellent resource- and space-saving characteristics^22^. Even on the Earth, the microgravity field experiments on AM have been conducted based on parabolic flight experiments in the United States, China, and Germany^23^.

The aforementioned research efforts related to microgravity AM have revealed that microgravity environments are not conducive for the fabrication process. In microgravity AM, fixing the material on the stage is challenging, and the residual failures in the deposit cannot be ejected because of the absence of buoyancy forces. Several AM studies have attempted to realize dense material supply and eliminate micropores in the deposit, even under 1 G conditions^24^. Based on the reported results, we expect that several evaluation indices will be improved under 1 G conditions. Thus, this conclusion serves as the motivation for the development of a novel AM technology that leverages gravity levels greater than 1 G. In this regard, the high-gravity material extrusion (HG-MEX) system is a specialized facility designed to operate under high-gravity conditions, thus creating new opportunities for commercial and industrial applications. HG-MEX can potentially address certain challenges pertaining to AM in space and microgravity conditions. Thus, it can play a crucial role in meeting the need for efficient and reliable manufacturing techniques demands and advancing AM in space and microgravity environments.

**Figure 1:**
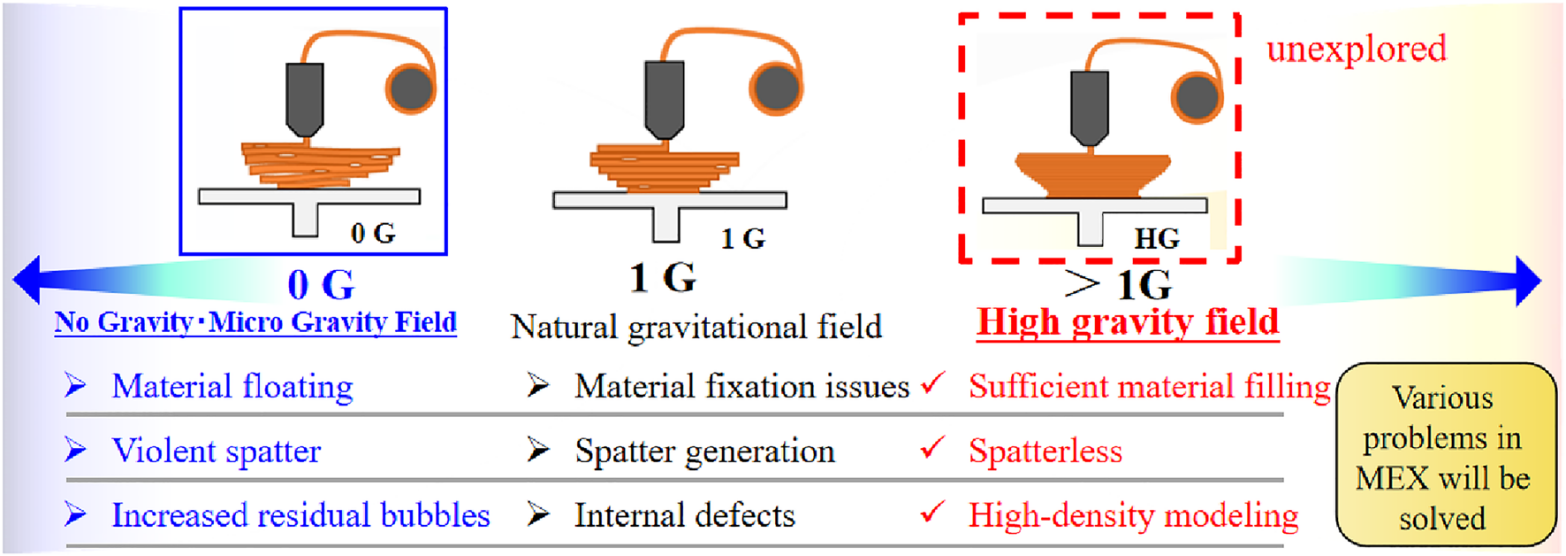
Differences in the MEX method due to changes in gravity.

This study focuses on material extrusion (MEX), which is one of the most widely used processes in metal AM. In the MEX process, a thermoplastic material is melted and extruded through a nozzle to create an object^25–27^. MEX can produce parts with adequate accuracy and quality^28–30^. The flexibility of MEX allows for the production of complex geometries and customized products, thus rendering it a valuable tool for prototyping and small-batch production^31–33^. In the context of MEX, gravity could potentially have several effects on the process^34–38^. Figure 1 presents differences in the MEX method due to changes in gravity.

In this study, an HG-MEX system was developed with a maximum output of 32 G (1 G = 9.81 m/s^2^), and the effects of high gravitational acceleration on the process and products were evaluated. Although utilization of the AM process in microgravity has attracted significant attention as a possible production method in space, no research has been conducted to actively apply high acceleration to the MEX process. High gravity may endow the MEX with various positive effects. Therefore, in this proposal, gravitational acceleration is regarded as a controllable factor that has considerable potential for improving the MEX process. In this study, experiments were conducted using various gravitational accelerations from 1 to 32 G, and the results demonstrate that the high-gravitational acceleration improves the MEX accuracy performance of the fabricated parts.

## High gravity in MEX

### Practical applications of centrifugal acceleration

To design an HG-MEX system, each component must be carefully considered to ensure efficient and effective brewing. Neglecting any one of these components can lead to various issues, including negative impacts on the quality and efficiency of the process.

**Figure 2:**
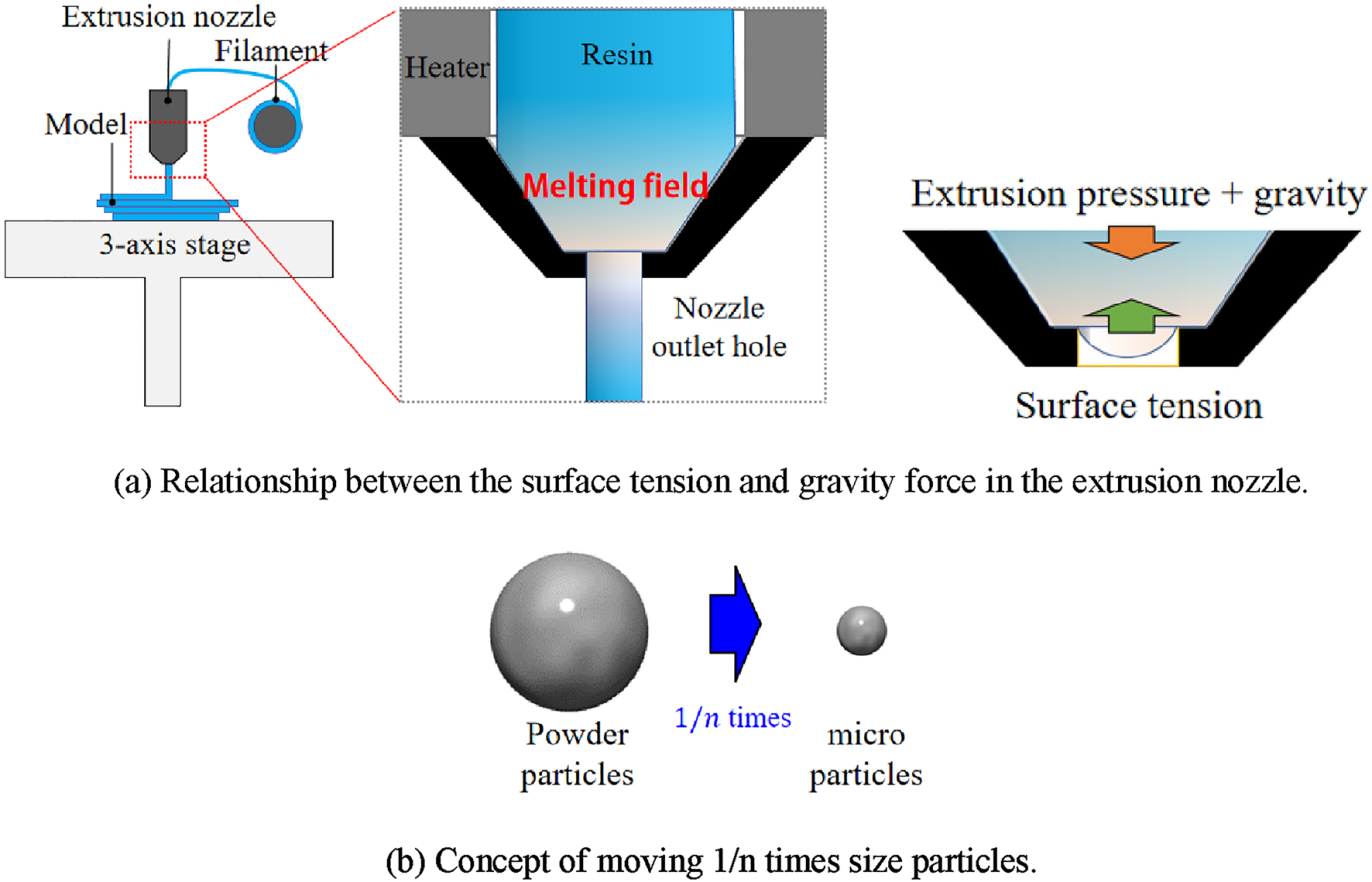
Material characteristic during MEX.

Centrifugal force is the apparent force that acts on an object moving in a circular path, directed away from the center of rotation. It is an inertial force arising from the object's motion, rather than any physical interaction. In a rotating system, centrifugal force is the outward force that pushes objects away from the center of rotation. This force is generally leveraged in industrial processes, such as for the separation of solid particles from liquids or gases in centrifuges. Figure 2 presents the relationship between the surface tension and gravity force in the extrusion nozzle.

To enable material ejection from the outlet hole, surface tension must be overcome. Surface tension is particularly strong in small-diameter holes. The modeling resolution in MEX depends on the diameter of the nozzle outlet hole. If the outlet hole diameter is extremely small, the material cannot be released owing to surface tension, as shown in Fig. 2a. Eötvös number (${\mathrm{E}}_{\mathrm{o}}$) is used as an index to evaluate whether the material can be extruded; it is defined by the following equation.

1 $${\mathrm{E}}_{\mathrm{o}}=\frac{\Delta \rho g{L}^{2}}{\sigma },$$

where $\Delta\uprho$ is the density difference; $\mathrm{g}$ is the gravitational acceleration; $\mathrm{L}$ is the hole diameter; and $\upsigma$ is the surface tension force. ${\mathrm{E}}_{\mathrm{o}}$ decreases by a factor of 1/n^2^ as the hole diameter decreases by a factor of 1/n; consequently, the surface tension predominates, thereby preventing MEX from the nozzle. Moreover, surface tension can significantly influence the Eötvös number and material molding processes^39–43^. Essentially, the concept involves moving 1/n times-sized particles in a similar manner as the original particles. By doubling the rate of decrease for each force, the direction of the acceleration vector is rendered the same value as that for the original particle. Figure 2b presents the concept of moving 1/n times size particles.

Similar analogous rules can be considered even in solid-phase part. As the particle size decreases by a factor of 1/n, the electrostatic and fluid forces (i.e., area forces) decrease by a factor of 1/n^2^ proportionally to the surface area and cross-sectional area of the particle, respectively. The scale factors associated with n- or n^2^-times gravity have been studied, and these similarity rules are referred to as the “analogy of different gravitational fields,” as proposed by Dr. Koike^44,45^. The analogy rules can be considered in the same manner. By accounting for gravitational effects in the design and process, the impact of gravity can be investigated, and more accurate and reliable results can be obtained.

### Improvement in MEX quality

The effect of gravity on MEX is an important consideration in 3D printing, particularly in applications that require high precision or accuracy. Gravity affects the extrusion process by exerting a downward force on the material as it exits the nozzle, which can lead to different performance characteristics. By understanding and accounting for the effects of gravity, high-quality MEX objects can be produced even in challenging environments. This is particularly important for applications where the impact of gravity on the object’s overall quality and performance can be significant.

### HG-MEX methodology

In the proposed HG-MEX system, the resultant acceleration of the centrifugal and gravitational accelerations is applied perpendicular to the build surface. The MEX unit is subjected to rotational motion using a vertical axial type, and the resultant acceleration (${a}_{r}$) acting on the modeling surface is the gravitational acceleration (${\mathrm{a}}_{\mathrm{g}}$) and centrifugal acceleration (${\mathrm{a}}_{\mathrm{c}}$).

The centrifugal acceleration, ${\mathrm{a}}_{\mathrm{c}}$ [m/s^2^], is expressed as follows.

2 $$a_{c} = r_{s} \omega_{s}^{2} ,$$

where ${\mathrm{r}}_{\mathrm{s}}$ [m] is the distance from the center of the rotating body to the build surface and ${\upomega }_{\mathrm{s}}$ [rad/s] is the rotational speed. The rotation speed of $\mathrm{N}$ [min^−1^] is expressed as:

3 $$N=\frac{60{\omega }_{s}}{2\pi }=\frac{30{\omega }_{s}}{\pi } \left({\omega }_{s}=\frac{N\pi }{30}\right).$$

The resultant acceleration ${\mathrm{a}}_{\mathrm{r}}$ [m/s^2^] is expressed as:

4 $${a}_{r}=\sqrt{{{a}_{c}}^{2}+{{a}_{g}}^{2}},$$

where ${a}_{c}$ [m/s^2^] is the centrifugal acceleration and ${\mathrm{a}}_{\mathrm{g}}$ [m/s^2^] represents the natural gravitational acceleration (1 G) in the direction perpendicular to the Earth’s surface. In addition, the relationship between the resultant acceleration and rotational speed N [min^−1^] is expressed as follows.

5 $$N=\frac{2\pi }{60}\sqrt{\frac{\sqrt{{{a}_{r}}^{2}-{{a}_{g}}^{2}}}{{r}_{s}}}.$$

Based on these equations, the experimental device in this study was designed to achieve a rotational speed of 39.6 [rad/s] with a diameter turntable, thus yielding a maximum resultant acceleration of 32 G (|r|= 0.2 m; N = 378.3 [min^−1^]) to the MEX unit.

## Experiment setup

### Fabrication apparatus

**Figure 3:**
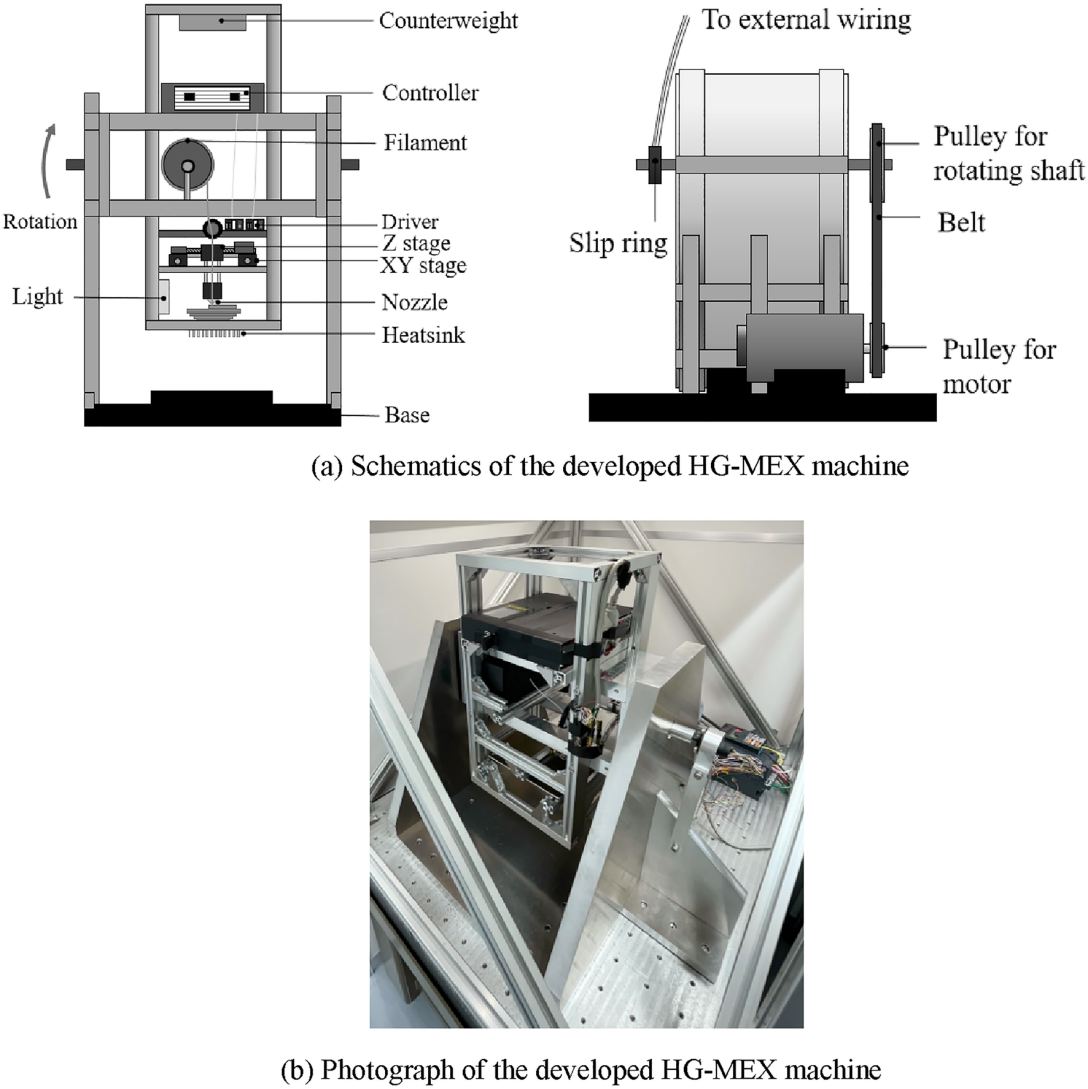
Schematics and photograph of the developed HG-MEX machine.

To compensate for the effect of high gravity on MEX, a specialized 3D printing system in space or other high-gravity fields must be developed. As many machine-tool builders already have excellent techniques for rotating large-mass objects at high speeds with high accuracy, a combined machine comprising a centrifuge and MEX unit is preferable from an economical viewpoint. Considering that the MEX unit needs to be installed on a rotation table, a compact and lightweight mechanism with a minimal number of components for the deposition process is desired. By rotating the centrifuge system, higher gravity conditions can be created. Figure 3 presents the 3D computer-aided design model and HG-MEX machine system developed in this study.

### Fabrication conditions and properties

In this research, the high-gravity MEX system was employed. By controlling the motor rotation speed, different levels of apparent gravity can be generated. Polylactic acid (PLA) is a popular thermoplastic material used in 3D printing^46,47^. The mechanical properties of the PLA filament used in this study are listed in Table 1.

**Table 1:** Mechanical properties of PLA filament.

Parameter	Value	Unit
Diameter	1.75	mm
Melt temperature	190–230	℃
Density	1.24	g/cm^3^
Tensile strength	60	N/mm^3^
Tensile modulus	3600	MPa
Poisson’s ratio	0.35	
Specific heat	2040	J/kg·K
Coefficient of thermal conductivity	0.231	W/(m K)
Coefficient of thermal expansion	1.999 × 10^–6^	
Thermal diffusivity	0.205	mm^2^/s

The MEX conditions can affect the properties of the fabricated parts in various ways, and adjusting these conditions can help achieve the desired properties^37,48^. Thus, herein, experiments with different gravity levels were designed by selecting an appropriate motor control suiting the gravity conditions and objectives of this study. The PLA filament was obtained from the manufacturer Voxelab. Further, 3D printing was performed under an ambient temperature of 25 ℃, with an extrusion speed of 10 mm/s. A nozzle temperature of 200 ℃ and bed temperature of 50 °C were set for MEX. The material was allowed to flow out naturally from the nozzle and naturally accumulate. The nozzle, a key component in fluid systems, is responsible for controlling the flow of fluids. The nozzle size used in this study was 0.4 mm. The filament was fed into the extruder and melted by the heated nozzle during the MEX process.

## Results and discussion

### Different gravity MEX

In MEX, by controlling the motor rotation speed, we simulated various gravity conditions, ranging from 1 to 32 G. By setting up a camera next to the MEX unit, the MEX process was captured. Note that when the material extrudes from the nozzle in a MEX 3D printer, it undergoes a series of physical and chemical changes as it is heated, melted, and cooled to form a solid object. The process of a material extruding from a nozzle in an MEX 3D printer is complex and precise.

### Evaluation for fabricated products

**Figure 4:**
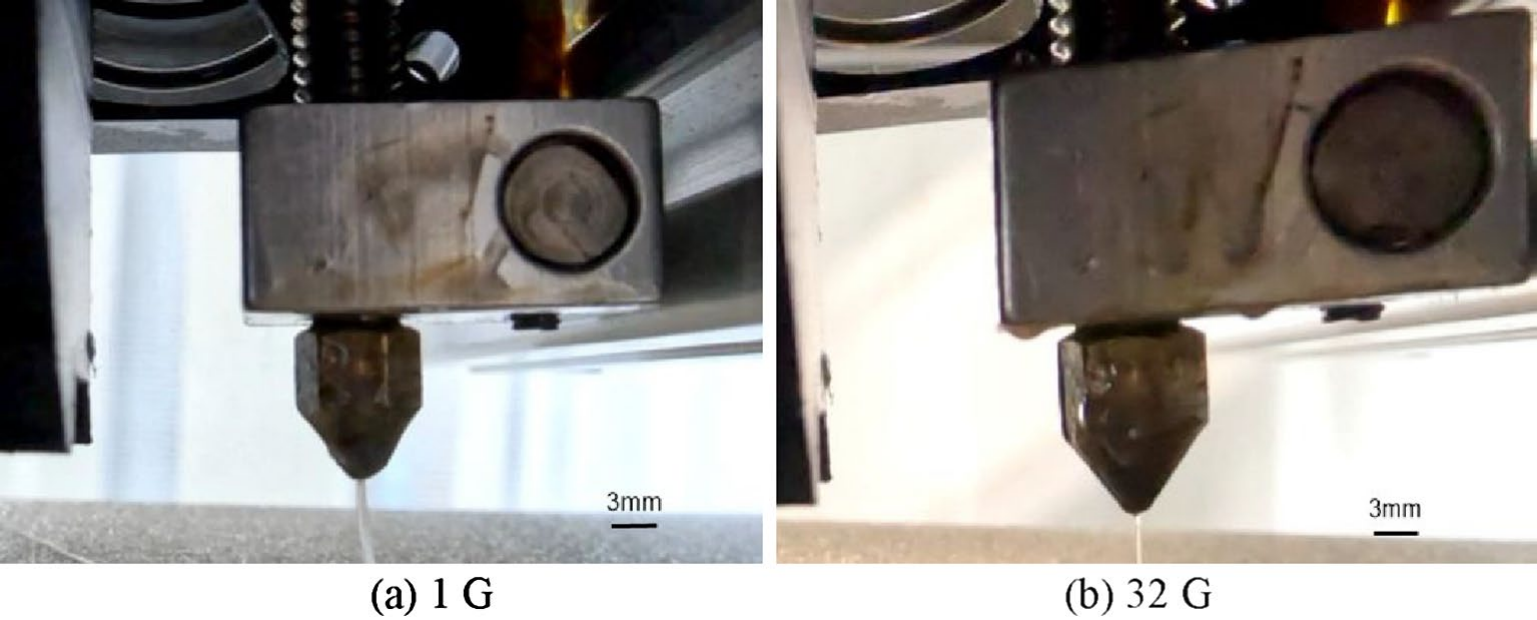
MEX under different gravity conditions.

As the melted material is deposited, it cools and solidifies, thereby forming the object. Considering the case wherein the material is directly extruded, we compared the effects of different gravity states on the extrusion process. The process of MEX in AM is influenced by the gravitational force. When the printer is operating in high-gravity conditions, the force and factors of gravity can cause the extruded material to behave differently than it would in 1 G conditions. In high-gravity conditions, the material may be pulled downward more strongly, thus resulting in thinning and stretching. This causes a decrease in the size of the extruded material and affects the overall quality of the object. Different gravity conditions result in different performance of the MEX. Figure 4 presents the results of MEX under different gravity conditions with a nozzle size of 0.4 mm.

As the material extrudes from the nozzle, it tends to maintain a cylindrical shape owing to surface tension and its properties. The surface tension causes the surface of the material to pull inward, i.e., toward the center of the cylindrical shape. By focusing on the area where the material extruded from the nozzle, we found that different gravity conditions resulted in different performance.

**Figure 5:**
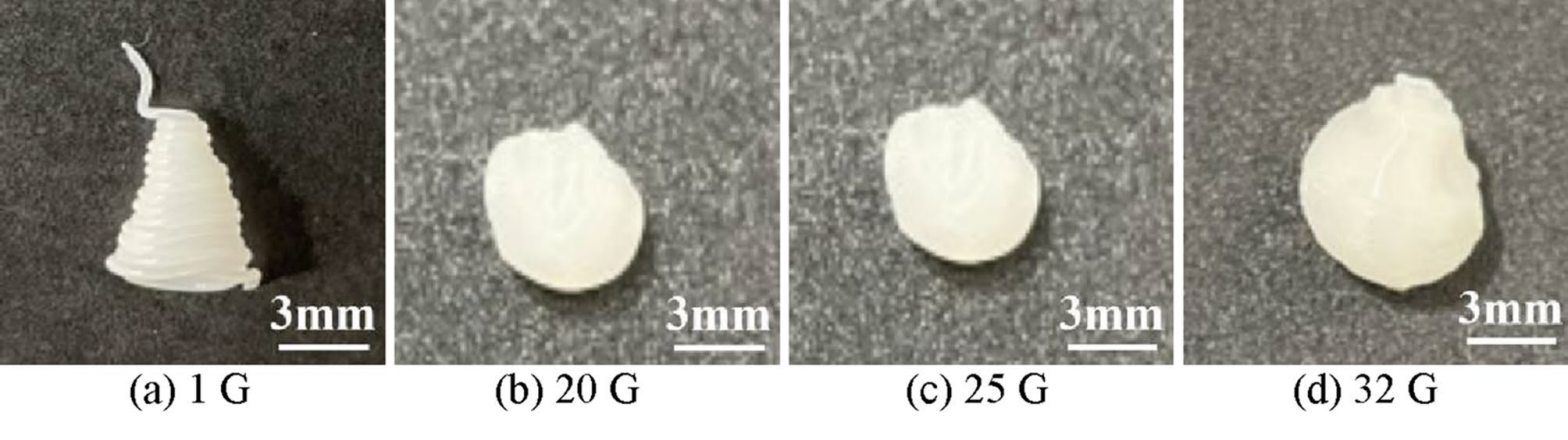
Overall view of the MEX with a nozzle of size 0.4 mm.

By viewing from the place of PLA just extrusion out from the nozzle, the MEX process is almost similar, by view the line size width, high-gravity field (20 G, 25 G, 32 G) line size is thinner than 1 G. Figure 5 presents an overall view of the MEX achieved with a nozzle size of 0.4 mm.

**Figure 6:**
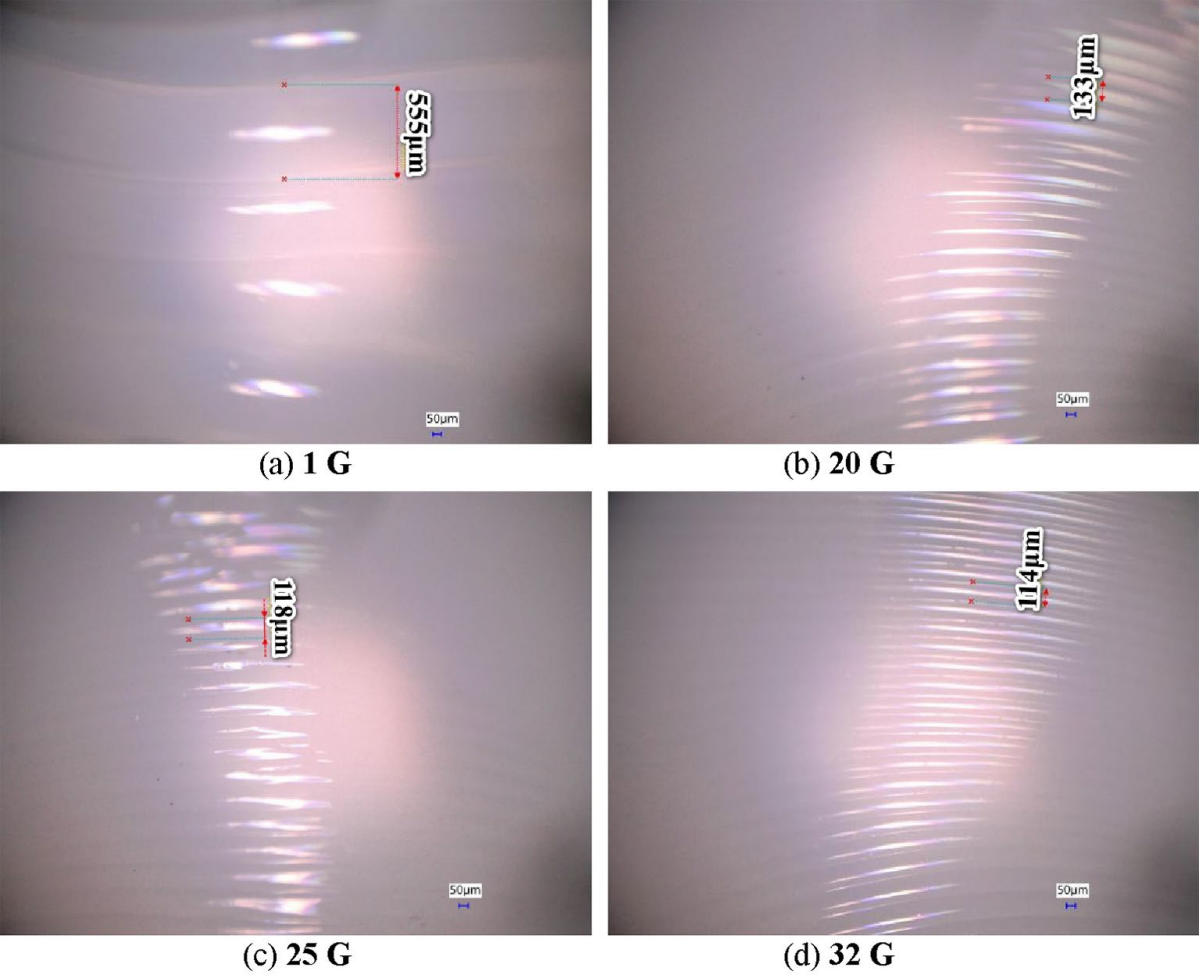
Influence of different gravity conditions on MEX, as view under a microscope.

MEX was achieved with a nozzle size of 0.4 mm. With this nozzle size, the printer can produce fine details and intricate geometries. Under different gravity conditions, the basic process of model formation is approximately identical, and the state of the model is also similar. The microscopic view of extrusion objects in different gravity conditions reveals the influence of different gravity levels on MEX; the observed differences are significant. Figure 6 presents the MEX view under different gravity conditions using a microscope.

**Figure 7:**
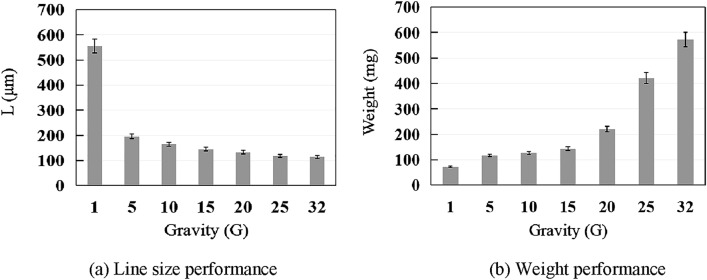
Performance of MEX under different gravity conditions.

In high-gravity PLA MEX, as the gravity increases, the line size (L) decreases, and the MEX performance improves compared with under 1 G. The line size (L) of the MEX under high-gravity conditions is significantly smaller than that under 1 G. Under a nozzle extrusion temperature of 200 ℃, we compared the MEX performances under different gravity fields. Gravity affects the behavior of a filament by pulling it downward, thus causing it to stretch or sag. Figure 7 presents the MEX performances under different gravity fields.

Figure 7a presents the line size performance. The MEX line size (L) under 1 G, 5 G, 10 G, 15 G, 20 G, 25 G, and 32 G is 555, 196, 165, 145, 133, 118, and 114 μm, respectively. The line size under the high-gravity condition of 32 G (114 μm) is smaller than that obtained under 1 G (555 μm). As gravity increases, the line size decreases. In particularly, from 1 to 32 G, the size decreases by approximately 79.5%, from 5 to 10 G, it decreases by approximately 15.8%, from 20 to 25 G, it decreases by approximately 11.3%, and from 25 to 32 G, it decreases by approximately 3.4%. Thus, as gravity increases, the ratio of decreased size reduces.

Figure 7b presents the weight performance of MEX under different gravity fields. The weight under 1 G, 5 G, 10 G, 15 G, 20 G, 25 G, and 32 G is 72, 117, 126, 143, 220, 421, and 573 mg, respectively. As gravity increases, the weight increases. In particular, from 1 to 32 G, the weight increased by a factor of approximately 6.96, from 15 to 20 G, it increased by a factor of approximately 0.54, and from 25 to 32 G, it increased by a factor of approximately 0.36. These results indicate that higher gravity simultaneously accelerates both the material flow and MEX. In particular, gravity influences the MEX and deposition accuracy of the object. By considering the influence of gravity and implementing strategies to mitigate its effects, the MEX characteristic and deposition accuracy of the object can be improved.

Under different gravity fields, the action of gravity possibly causes a relevant change in surface tension. Surface tension plays an important role in the behavior of the material as it extrudes from the 3D printing nozzle. The material extruded from the nozzle forms a bead of molten plastic. As the droplet of material cools and solidifies, its shape is influenced by the surface tension of the material. The surface tension causes the material to pull inward toward the center of the droplet. Thus, high gravity can lead to a more pronounced compression of the material, thereby resulting in thinner extrusion and potentially higher flow rates. Thus, a thinner extrudate and faster material flow are achieved under higher gravity. This thinner extrudate influence the geometry in AM processes. The combined effect of these factors lead to different performances under different gravitational field conditions. These characteristics are beneficial for achieving precise material deposition, particularly for intricate or fine details of the object. Thus, HG-MEX shows promise in enhancing the shape and flow velocity performance of fabricated parts.

## Conclusion

This study successfully developed a system capable of generating a high gravitational field in the MEX process for performing MEX. The effect of gravitational acceleration on the process and the fabricated deposit was experimentally evaluated. The increased gravitational forces can lead to a more pronounced compression of the material, essentially resulting in thinner extrusion and potentially higher flow rates. These results demonstrate the promising potential of high gravity in AM.

This study evaluated the gravity-driven positive effects on MEX, an AM. The experimental results indicate that high gravitational conditions can enhance the shape accuracy performance of the parts fabricated through MEX. Notably, the MEX showed significant improvements with a high gravitational field of 20 G or greater, which was particularly pronounced at higher recoating speeds. The line size and weight under 32 G decreased by approximately 79.5% and increased by a factor of 6.96 from that under 1 G, respectively. These results confirm the enhanced performance of MEX under high gravity, and demonstrate the applicability of HG-MEX.

The results suggest that the HG-MEX system offers distinct advantages without altering the material composition by leveraging high gravity. The increased gravity facilitates higher flow rates and enables the production of thinner extrudates. These characteristics are beneficial for precise material deposition, particularly for intricate or fine details of the object. Thus, HG-MEX holds great potential for addressing various challenges in MEX. In future research, we plan to explore even higher gravity conditions, beyond 32 G, and investigate more complex MEX scenarios. This will allow us to further uncover the unprecedented advantages of HG-MEX, such as defect-free fabrication and improved surface smoothness.

## Data Availability

The datasets used or analysed during the current study available from the corresponding author on reasonable request.
